# A study of the vernalization requirements of mountain rye (*Secale strictum syn. S. montanum*) may help explain low grain yields of perennial cereals compared to wheat

**DOI:** 10.1093/aobpla/plaf015

**Published:** 2025-03-19

**Authors:** Peter J Innes, Matthew T Newell, Keith G Pembleton, Richard C Hayes, Ando M Radanielson

**Affiliations:** Centre for Sustainable Agricultural Systems, University of Southern Queensland, 487-535 West Street, Toowoomba 4350, Australia; NSW Department of Primary Industries, Cowra Research and Advisory Station, Binni Creek Road, Cowra, NSW 2795, Australia; Centre for Sustainable Agricultural Systems, University of Southern Queensland, 487-535 West Street, Toowoomba 4350, Australia; NSW Department of Primary Industries, Wagga Wagga Agricultural Institute, PMB, Wagga Wagga, NSW 2650, Australia; Centre for Sustainable Agricultural Systems, University of Southern Queensland, 487-535 West Street, Toowoomba 4350, Australia

**Keywords:** Perennial grains, perennial cereals, intermediate wheatgrass, vernalization, dual-induction, wheat, mountain rye

## Abstract

Mountain rye (MR: *Secale strictum syn. S. montanum*) is a forage grass that is considered a candidate for perennial grain development in Australia. A greater understanding of the triggers for flowering would aid the integration of this dual-purpose perennial into Australian grazing and cropping systems. The objective of this experiment was to determine the effects of cold incubation (vernalization) periods of varying duration on the grain yield, biomass production, and phenology of MR, compared to a dual purpose annual winter wheat cultivar (AW: *Triticum aestivum* L), and the perennial intermediate wheatgrass (IWG: *Thinopyrum intermedium* (Host) Barkworth & D.R. Dewey). Plants were grown in pots to a three leaf stage, before being subjected to vernalization treatments of varying length (0, 3, 5, 7, 9, and 11 weeks) using a growth chamber set to 5°C and 10 hour lighting. The plants were then transferred to a glasshouse set to 22°C and a minimum of 10 hour lighting. Glasshouse daylengths increased as the season progressed from winter into spring and summer. Exposure at ≤5°C for 5 weeks in MR optimized reproductive development, compared to approximately 3 weeks for winter wheat and 11 weeks for IWG. Both MR and IWG appear to have a dual induction requirement, needing a period of cold exposure and short days, followed by daylengths of 12–13 hours for MR, and at least 13 hours for IWG, to optimize their grain production potential. The observed higher vernalization requirement of MR, compared to AW, helps delineate the grain production potential of the tested germplasm in current wheat-growing areas of temperate Australia. Reproductive tiller percentages were much higher and developed earlier in AW compared to the perennial cereals. Improving the ratio and timing of reproductive tillers development in perennial cereals should be a target if grain yields are to be improved.

## Teaser

Most cereal grains are annuals that need replanting each year. Perennial cereals are being developed that can be grazed and harvested for grain yearly but can persist for multiple years. Mountain rye and intermediate wheatgrass are being evaluated as dual-purpose crops in Australia. As they are both derived from cool climate grasses, they require vernalization, a period of cold and short days, followed by warmer long days. These requirements are reasonably well understood for intermediate wheatgrass, but not for mountain rye. An experiment comparing the chilling requirements of mountain rye, intermediate wheatgrass, and annual wheat was undertaken in Cowra. The results indicated mountain rye requires more vernalization than annual wheat, but less than intermediate wheatgrass. This information is needed to help guide future plant development and identify geographical areas suitable for perennial grain production.

## Introduction

Introducing perennial cereals into Australian farming systems has many potential advantages including reduced tillage and improved soil organic matter accumulation (Kim *et al.*, 2022; Tang *et al.*, 2023), reduced nutrient leaching (Culman *et al.*, 2013), and potential production benefits from reduced farm inputs and extra grazing for livestock (Bell *et al.*, 2010; M. Newell *et al.*, 2025). A range of perennial cereals, potentially suitable for integration into Australian farming systems, have previously been evaluated (Hayes *et al.*, 2012, 2018; Larkin *et al.*, 2014). In those initial evaluations, mountain rye (MR: *Secale strictum syn. S. montanum*) was highlighted as a species with superior persistence compared to other perennial grain crops and is a candidate for *de novo* domestication, similar to the development of intermediate wheatgrass (IWG:*Thinopyrum intermedium* (Host) Barkworth & D.R. Dewey) to produce the grain crop Kernza™ (Bajgain *et al.*, 2022). As a forage species, MR is best adapted to cooler areas of south-eastern Australia (Bishop *et al.*, 1987; Oram, 1996), but has recently been found to have grain characteristics suitable for a range of food products (Baker *et al.*, 2021; Sinclair *et al.*, 2021).

In both annual and perennial temperate cereals, vernalization requirements and photoperiod sensitivity are important considerations for optimal adaptation to different growing environments and grain production. Vernalization is a requirement in some plants for exposure to cold temperatures to induce flowering (Chouard, 1960; Heide, 1994). The majority of temperate perennial grasses require dual induction to initiate the transition from vegetative to reproductive growth (Heide, 1994). Cool temperature and short daylength (primary induction) promote the development of inflorescence primordia in the meristem. The transition to warmer temperatures and longer daylength (secondary induction) promotes stem elongation and inflorescence development. There is variation in the induction requirements between species and within ecotypes of the same species (Fjellheim *et al.*, 2014). In wheat (*Triticum aestivum* L) this has been traced to the VRN1 gene system with orthologue genes across all three genomes (Eagles *et al.*, 2010; Sehgal *et al.*, 2024). Winter wheat can have substantial vernalization requirements, while spring wheat is insensitive. Photoperiod genes (PPD complex in wheat) promote the transition to flowering, and in sensitive temperate species, are triggered by long-day thresholds (Sehgal *et al.*, 2024). In rye (*Secale* spp.), short days may influence the expression of the VRN2 gene and delay the transition to reproduction (Cox *et al.*, 2002). An understanding of these requirements for candidate perennial cereal species will be important to allow integration of these findings into process-based crop models, to help identify potential zones of adaptation, and to inform plant breeding programs.

A commonly grown winter wheat (AW) in south-eastern Australia, and two candidate perennial cereals (MR and IWG) were included in the experiment. The winter wheat was known to have a vernalization temperature requirement of 5-10°C, with a duration of 8 days or more, and a subsequent photoperiod requirement of >10 hours (Matthews *et al.*, 2019; Bloomfield *et al.*, 2023). The other comparison species, IWG, has been grown as a perennial grain in the higher latitudes of the northern hemisphere. For optimal grain production in these environments, IWG has been found to have a vernalization temperature requirement of 4–5°C, for a duration 5–7 weeks, and a day length of <11 hours increasing to >13 hours for secondary induction (Ivancic *et al.*, 2021; Locatelli *et al.*, 2022, 2023).

There are little data available regarding the vernalization requirements of MR for flowering and grain yield optimization. The annual cereal rye (*Secale cereale* L), a close relative of MR (Gruner *et al.*, 2021), is known to require a vernalization temperature of 5–10°C, for a duration of 6–8 weeks, plus a subsequent photoperiod of greater than 14 hours for optimal grain production (Schlegel, 2013). The hypothesis was that MR would have higher vernalization requirements than AW, but lower than IWG and that all species would have reduced grain yields if not vernalized sufficiently. Further, it was proposed that insufficient vernalization would reduce the ratios of reproductive to vegetative tillers and increase the aboveground vegetative biomass of each species.

## Methods

### Germplasm

The population of MR used has shown potential as a perennial forage species in Australasia (Oram, 1996). It exhibits a range of desirable grain attributes for a diversity of end uses (M. T. Newell *et al.*, 2021). The IWG plant material used (CPI-148055) was previously evaluated in Australia (Hayes *et al.*, 2012; Larkin *et al.*, 2014) and was from an early-generation breeding population obtained from the Land Institute (Salina, KS) in 2009 (previously under the breeder code IWG 3182). Both MR and IWG populations displayed long maturity times and an ability to produce grain in Australian environments. The AW used was ‘EGA_Wedgetail’, a common industry benchmark winter wheat cultivar.

### Glasshouse conditions

The experiment was conducted in the Glasshouse facilities of NSW Department of Primary Industries Cowra Agricultural Research and Advisory Station, NSW, Australia. Plants were grown in black plastic pots, dimensions 190 mm high × 200 mm diameter, each containing approximately 2.8 L of a proprietary potting mix (Scotts Osmocoat® Premium). Treatments were replicated four times giving a total of 72 pots. Prior to sowing, the seed of each entry was cleaned and graded to provide good quality seed for planting. In May 2023, five seeds from the same entry were sown in each pot and thinned to one plant once one leaf had fully emerged. The winter wheat was sown five days after the perennial cereal species to ensure that each species was exposed to the vernalization treatments at a similar growth stage. Each pot received a complete nutrient solution (Thrive®: 25%N, 5%P, 8.8%K, 4.6%S, 0.5%Mg, 0.18%Fe, 0.01%Mn, 0.005%Cu, 0.001% Zn, diluted to 2g L^-1^ H_2_O) once every 2 weeks, after the seedlings had fully established. Pots were placed on flood and drain benches and watered twice daily using an automated irrigation system. Temperatures in the glasshouse were controlled at an average 22.5°C (SD 2.6, min. 15, max. 28) throughout the experiment. The photoperiod was extended using auxiliary horticultural lighting (Hort. L2 Module, Samsung) to a minimum of 10 hours during the day period in the glasshouse. The photoperiod increased throughout the experiment as the natural day length progressed with the season (Table 1).

**Table 1.** Glasshouse daylengths vs. elapsed days

Date	19th Jun.	13th Jul.	7th Aug.	1st Sep.	26th Sep.	21st Oct.	15th Nov.	10th Dec.	4th Jan.
Elapsed days	0	25	50	75	100	125	150	175	200
Daylength (h)	10.0	10.1	10.7	11.4	12.3	13.1	13.8	14.3	14.3

### 1.2.3 Vernalization treatment

Treatments included five periods of vernalization (0, 3, 5, 7, 9, and 11 weeks). Plants assigned to the zero vernalization treatment remained in the glasshouse for the entirety of the experiment. Plants undergoing vernalization were all transferred to a growth chamber on the same day, by which stage all plants were at the three-leaf stage. The average temperature within the growth chamber was 4.9°C (SD 0.4) with 10 hours of lighting (800 µmols m^−2^s^−1^ photosynthetic photon flux density) similar to the conditions used by Locatelli *et al.* (2022). Once each period was completed, pots within that treatment were returned to the glasshouse. Pots were re-randomized within each replicate group as they entered the growth chamber and then returned to the glasshouse.

### Measurements

Tiller numbers and phenological development were assessed at 3—4-week intervals, from 19th June 2023 to 6th February 2024 (1–233 days after the start of vernalization treatments). As plants transitioned to reproductive development, a count of reproductive vegetative tillers was completed. Plants were assessed every 3–4 days once head emergence was initiated and anthesis dates recorded. When plants reached full maturity total head numbers were counted and seed heads were removed for drying, weighing, and threshing. All other plant material was removed to 50 mm above soil level and the combined stems and leaves, along with any senesced material, were dried and weighed.

### Data analysis

The experimental design was a randomized complete factorial with two factors: species (AW, IWG, MR) and vernalization treatment (0, 3, 5, 7, 9, and 11 weeks). The treatment factor included the effects of varying times in the growth chamber and glasshouse, including temperature, thermal time (Bonhomme, 2000), and photoperiod differences. The experimental unit was each pot containing one plant. The response variables (biomass, grain, tiller numbers) were analysed by ANOVA with the R programming language version 4.3.1 (R-Core-Team, 2021) using the *aov* command. ANOVA assumptions of normality were checked visually by plotting the residuals. When ANOVA identified an effect at *P* ≤ 0.05, Fishers LSD (95% confidence level) was used to separate the means using the *LSD.test()* function supplied by the agricolae library for R (De Mendiburu *et al.*, 2020). Species were analysed together for main and interaction effects (Table 2). Analysis was also done at individual species level. The individual species analysis only produced significant results for AW yields and IWG reproductive tillers percentages (results not shown).

**Table 2.** Species and treatment effects on yields and tiller numbers.

Species	Treatment	Total DM	Grain	TKW	Reproductive Tillers	Total Tillers
	*weeks*	*g/plant*	*g/plant*	*g*	*%*	*count*
**Annual Wheat**	**0**	51.0 a	17.8 b	29.7	59.1 bc	36.8 ef
	**3**	27.1 de	15.4 bc	38.3	50.2 cd	20.5 f
	**5**	20.3 e	11.6 c	38.5	43.8 cd	17.3 f
	**7**	21.1 e	11.6 c	36.9	44.7 cd	17.8 f
	**9**	29.7 cde	15.4 bc	31.2	73.6 b	20.8 f
	**11**	50.2 a	23.0 a	35.8	97.9 a	19.0 f
**Mountain Rye**	**0**	40.6 abcd	0.1 d	14.2	6.8 f	120 a
	**3**	39.0 abcd	0.3 d	10.2	5.6 f	78.3 bc
	**5**	44.3 abc	0.8 d	13.6	15.5 ef	83.0 b
	**7**	44.2 abc	1.0 d	10.6	19.4 ef	78.0 bc
	**9**	47.4 ab	1.1 d	10.8	35.0 de	57.8 cde
	**11**	42.6 abcd	1.6 d	11.7	30.8 de	60.5 cd
** Intermediate Wheatgrass**	**0**	51.3 a	0.0		0 f	53.3 de
	**3**	41.8 abcd	0.0		0 f	73.3 bcd
	**5**	35.5 abcde	0.0		0 f	68.3 bcd
	**7**	26.5 de	0.0		0.3 f	61.0 bcd
	**9**	28.8 cde	0.0		0.4 f	55.5 de
	**11**	31.5 bcde	0.0		5.5 f	66.5 bcd
** Species effect**	***P* value**	*0.02*	*<0.01*	*<0.01*	*<0.01*	*<0.01*
** Vern effect**	***P* value**	*0.01*	*0.01*	*0.02*	*<0.01*	*<0.01*
** Species:Vern**	***P* value**	*0.01*	*0.04*	*0.33*	*0.03*	*<0.01*
	**LSD (*P* = .05)**	*16.61*	*5.03*		*21.0*	*22.5*

Treatments are the number of weeks of vernalization at 5°C. DM = Dry Matter, TKW = Thousand Kernel Weight. Grain and TKW were analysed without IWG as IWG produced no grain. Reproductive tiller numbers were recorded at the time of harvest. Total Tillers are the maximum recorded tiller count. Results are the means of the four replicates. Where results share a lower case letter the effects were not significantly different.

## Results

The AW plants were harvested from November 2023 to January 2024 as the grain matured. The 3–9 weeks vernalization treatments matured earliest. The MR matured between December 2023 and January 2024. The earlier MR harvests were in the 5–9 weeks of vernalization treatments. By March 2024, IWG had produced no grain at all, regardless of the vernalization treatment. The main and interaction effects of the species and vernalization treatments on the grain yields, biomass, and tiller numbers of each species are presented in Table 2. There was a 16-fold increase in MR grain production, from a low of 0.1g/plant at 0 weeks of vernalization treatment to a high of 1.6 g/plant at 11 weeks of treatment, with an 8-fold increase after just 3 weeks of treatment. IWG biomass weights decreased by 39% from a high of 51.3 g/plant at 0 weeks of treatment to a low of 31.5 g/plant at 9 weeks of treatment. The effect of vernalization treatment on MR reproductive tillers was not significant, but an increase in reproductive tillers can be observed after 5–7 weeks of treatment. There were no reproductive tillers evident for IWG until 7 weeks of vernalization treatment but none of these proceeded to grain maturity. However, when analysed at the individual species level, there was a significant increase in the IWG reproductive tiller percentage at 7 weeks (analysis not shown).

### Phenology: Zadoks growth scores during and after treatment periods

**Figure 1.**
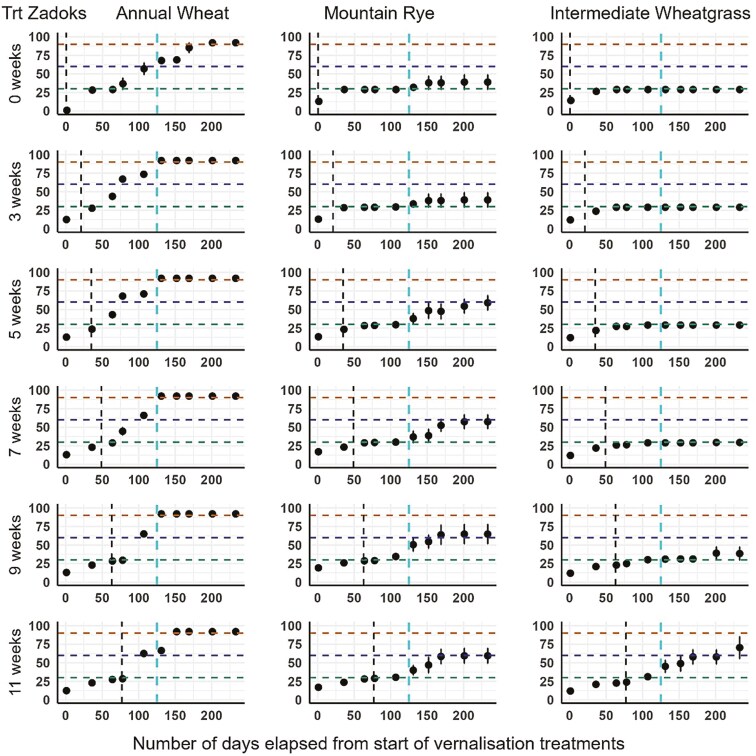
Zadoks growth scores vs. days from start of each vernalization treatment. Points are the mean Zadoks score of the four replicates for each treatment. Error bars represent standard error of the mean. Points below the lowest (green) horizontal dashed line are the vegetative stage, between the middle (blue) and green lines are early reproductive stages, and above the middle (blue) line are the grain development stages. The top (orange) horizontal line indicates grain maturity. The first vertical dashed line is the point where the plants were moved from the growth chamber to the glasshouse. The second vertical dashed line at 125 days is the point where glasshouse daylengths increased to > 13 hours (Table 1).

Annual Wheat reached grain ripe Zadoks stage (>90) for all treatments, but was slower for the control, compared to the vernalized treatments (Fig. 1). MR progressed to the grain development stage (Zadoks > 60) after 5 weeks or more of vernalization. The IWG only progressed beyond the vegetative stage (Zadoks > 29) when exposed to 9 or more weeks of vernalization, but no plants progressed to the grain ripe stage.

**Figure 2.**
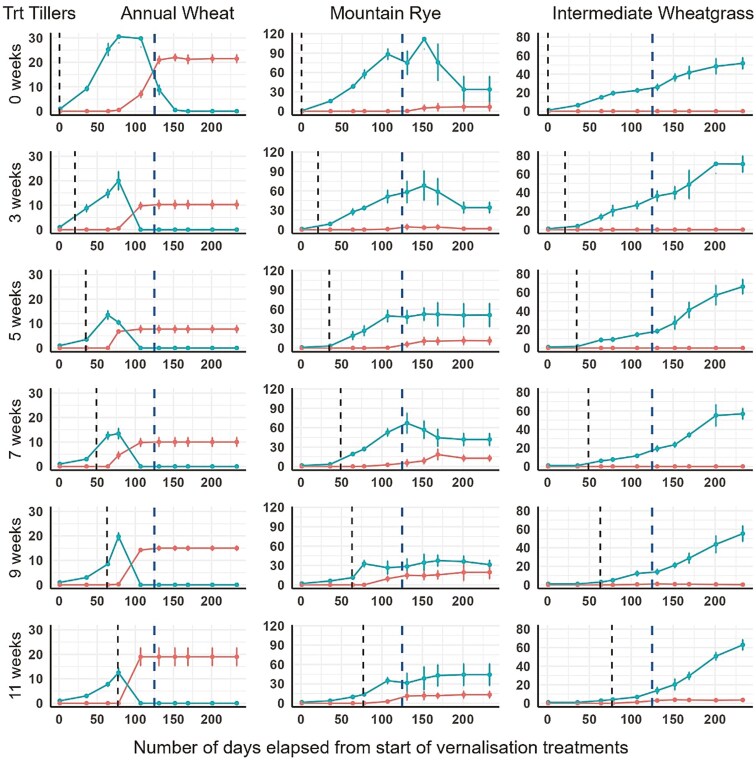
The number of vegetative and reproductive tillers (y-axis) of AW, MR, and IWG after different periods of vernalization. The x-axis is days elapsed from the treatment starts. Vegetative tillers are first tillers to develop (green dots and lines), reproductive tillers develop later when reproduction has been triggered (orange dots and lines). ‘Trt’ is the treatment time in weeks at 5°C. Error bars are standard error of the mean. The first vertical dashed line is the point where the plants were moved from the growth chamber to the glasshouse. The second vertical dashed line at 125 days is the point where glasshouse daylengths increased to >13 hours.

Annual wheat (Figure 2) had the highest number of vegetative tillers in the 0 and 3 weeks treatment groups. Vegetative tiller numbers in MR were suppressed by 5 weeks or more of vernalization, with a corresponding increase of reproductive tillers. Reproductive tiller development in IWG was only apparent within 9 and 11 weeks treatment groups and occurred after the end of the vernalization treatment periods (i.e. after 77 days).

## Discussion

It was hypothesized that MR would require a longer period of vernalization than AW and less than IWG. The results of this experiment support the hypothesis, indicating MR may require 1–2 extra weeks of cold exposure than AW, plus a 12–13 hour day length for secondary induction (see Fig. 1), compared to around 11 hours for AW. The IWG seemed to need an even longer cold exposure, of at least 9 weeks, and a 13-hour day length for secondary induction. The experiment was not specifically designed to test the effects of daylength on floral initiation as that would have required daylengths in both the growth chamber and glasshouse to be controlled (e.g. be equal) for up to 11 weeks. However, other researchers have noted that in the case of perennial cereals, calendar days (and thus daylengths) can be a better predictor of secondary induction than thermal time (Duchene *et al.*, 2021; Barriball *et al.*, 2022) The 7–11-week treatment plants had shorter initial daylengths in this experiment, remaining at 10 hours photoperiod duration in the growth chamber, while those moved to the glasshouse (3–5-week treatments) experienced 11.5 hours daylengths by 11 weeks (Table 1). The AW would have been most affected by the longer glasshouse daylengths, having a relatively shorter day length requirement to progress to reproduction, while MR and IWG appear to have at least a 12-hour requirement. Thus, the photoperiod differences between the growth chamber and glasshouse would be less important for MR and IWG, as the longer initial glasshouse daylengths should not have triggered secondary induction in these species. Additionally, it was proposed in the current study that all species would have reduced grain yields if not vernalized sufficiently. For MR, this was confirmed as grain yields were optimized after five weeks of vernalization treatment. The results for AW and IWG were inconclusive. The 0 weeks vernalization treatment for AW produced a higher grain yield than the 3–9 week vernalization treatments. The higher thermal time experienced by the 0 weeks treatment possibly increased the AW grain yield, although lack of vernalization in AW is known to extend the thermal time to reproduction (Bloomfield *et al.*, 2023). It is also known that a sufficient number of short daylengths may override the need for low vernalization temperatures in some plants (Heide, 1994), but it is not known this was the case for AW. The IWG did not progress to grain development for any of the treatments. It is possible that the daylengths in the growth chamber and glasshouse did not match the daylengths required for the phenology to proceed to flowering. Locatelli *et al.* (2022) in a similar experiment grew their plants to 3 leaves seedling stage with a mixture of 8 hours of winter daylight plus 8 hours of low-intensity incandescent light per day, resulting in a shorter period of darkness per day compared to our experiment that used 10 hours of light and 14 hours of darkness per day. There can be an interaction between day length and temperature for some dual induction plants (Heide, 1994). Also, if the time between vernalization and the requisite daylength for induction is too long (and subject to higher temperatures), plants may de-vernalize (Locatelli *et al.*, 2022). This may not be the case for IWG, but if it was, it could explain why the plants that remained longest in the growth chamber (at low temperature with less devernalization risk) proceeded to flowering. It is also possible that a stress factor, such as a heat episode, affected canopy-level reproductive organs. Recorded glasshouse temperatures ranged as high as 28°C during early October. It is known that reproductive development in IWG is susceptible to higher temperatures (Ivancic *et al.*, 2021), but the critical temperature levels and durations are unknown. Farooq *et al.* (2011), in their review of heat stress in wheat, reported damaging temperatures occurring at 21.4 ± 2.33°C for the terminal spikelet stage and 32 ± 1.74°C for the flowering stage.

It was also expected that the ratio of reproductive to vegetative tillers would increase with sufficient vernalization. The ratio of reproductive to vegetative tillers in MR increased after 3-7 weeks of treatment, possibly when optimal vernalization was achieved. Reduction in vegetative tiller numbers after vernalization has been noted in previous studies (Steinfort *et al.*, 2017). The IWG only produced reproductive tillers when subjected to a 7–11-week vernalization treatment, making it difficult to draw any conclusion about the effects of vernalization on the tiller dynamics of IWG in this experiment, although there was a significant increase in the percentage of reproductive tillers at 7 weeks. Locatelli *et al.* (2022) observed an increase in spikes in IWG from 3 to 7 weeks of vernalization treatment and inferred a concurrent decrease in total tiller numbers, due to competition for assimilates during the stem elongation to flowering stages (Colvill *et al.*, 2008). The proportion of fertile tiller numbers is an important consideration for perennial cereals and has been found to be a good predictor of grain yield (Fernandez *et al.*, 2020). However, perennial cereals need to retain a proportion of vegetative meristems to ensure the plant continues growing into the next season (Fjellheim *et al.*, 2014). Thus, there is a trade-off between reproductive tiller development and vegetative tiller retention in perennial cereals.

It was proposed that vernalization would decrease the vegetative biomass of each species. There was no apparent trend in biomass for either AW or MR. Therefore, the hypothesis of decreasing total biomass after sufficient vernalization treatment was not confirmed for these species. However, there may have been an influence from the different thermal time accumulations during the vernalization treatment periods. In such experiments, it is almost impossible to isolate the effects of vernalization and thermal time accumulation as, by definition, vernalization period reduces thermal time accumulation. The 11-week vernalization treatment had less thermal time accumulated at the time of harvest than the other treatments. The difference in thermal time accumulation between vernalization treatments was greater for the earlier harvested plants, such as AW harvested in December 2023, and MR harvested in January 2024.

## Conclusion

In this study, a vernalization period of 5 weeks at 5°C optimized the grain production potential of MR, compared to 3 weeks for AW. The AW became fully reproductive when a 10-11 hour daylight period was experienced, while for MR it was 12–13 hours, and for IWG it was approximately 13 hours. Both MR and AW produced a higher number of vegetative tillers when there was less than 3 weeks of vernalisation. These results help in the selection of viable cropping regions and management strategies for MR as a dual-purpose perennial grain. The results also highlight the high vernalization requirements of MR and IWG in relation to AW. The reduction of these requirements should be a priority for plant breeders if MR and IWG are to be viable dual purpose cropping options at lower latitudes. Of note is the much higher ratio of reproductive tillers to vegetative tillers in AW, post-vernalization, compared to MR and IWG. This should also be considered by plant breeders if grain yield to biomass ratios are to be improved in MR and IWG. In future experiments, a closer monitoring of both temperature and light exposure (e.g. loggers in individual pots), and varied photoperiods during vernalization, may help disentangle the three-way effects of vernalizing temperature, photoperiod, and thermal time on reproductive development in MR and IWG. Field experiments in contrasting environments would also be useful, to further define the interaction of latitude and seasonal temperatures with the dual induction requirements of MR and other perennial cereals, such as IWG.

## Data Availability

The datasets used to for the analysis presented in this article are available at: https://doi.org/10.26192/zwv51
